# Anatomical Variations of Cystic Ducts in Magnetic Resonance Cholangiopancreatography and Clinical Implications

**DOI:** 10.1155/2016/3021484

**Published:** 2016-05-25

**Authors:** Radha Sarawagi, Shyam Sundar, Sanjeev K. Gupta, Sameer Raghuwanshi

**Affiliations:** Deptartment of Radiodiagnosis, Mahatma Gandhi Medical College and Research Institute, Pillaiyarkuppam, Pondicherry 607 403, India

## Abstract

*Background*. Anatomical variations of cystic duct (CD) are frequently unrecognized. It is important to be aware of these variations prior to any surgical, percutaneous, or endoscopic intervention procedures.* Objectives*. The purpose of our study was to demonstrate the imaging features of CD and its variants using magnetic resonance cholangiopancreatography (MRCP) and document their prevalence in our population.* Materials and Methods*. This study included 198 patients who underwent MRCP due to different indications. Images were evaluated in picture archiving communication system (PACS) and variations of CD were documented.* Results*. Normal lateral insertion of CD at middle third of common hepatic duct was seen in 51% of cases. Medial insertion was seen in 16% of cases, of which 4% were low medial insertions. Low insertion of CD was noted in 9% of cases. Parallel course of CD was present in 7.5% of cases. High insertion was noted in 6% and short CD in 1% of cases. In 1 case, CD was draining into right hepatic duct. Congenital cystic dilation of CD was noted in one case with evidence of type IV choledochal cyst.* Conclusion*. Cystic duct variations are common and MRCP is an optimal imaging modality for demonstration of cystic duct anatomy.

## 1. Introduction

Anatomic variations of cystic ducts are common and frequently encountered during imaging. Failure to recognize some of the clinically important variants may lead to complication during surgical, endoscopic, or percutaneous intervention procedures [1]. Familiarity with the imaging appearance of cystic duct anatomy, its variant, and associated disease process helps in proper interpretation of the findings and helps in accurate diagnosis.

Noninvasive imaging technique that can delineate the cystic duct anatomy prior to any intervention procedure could be of great clinical significance. Nondilated cystic duct is difficult to visualize in USG. Proper visualization of the normal caliber bile duct in CT requires intravenous cholangiographic contrast media. MRCP is the optimal noninvasive imaging modality to delineate the anatomy of cystic duct and common bile duct.

Cystic duct is about 2–4 cm long and 1–5 mm in caliber which connects the neck of gall bladder to the common hepatic duct (CHD) to form the common bile duct (CBD). The point of insertion of the cystic duct into the CHD is variable. Most commonly it enters the CHD from the right lateral aspect [1]. It joins the CHD approximately halfway between the hepatic confluence and ampulla of Vater.

Different cystic duct variations are described in the literature based on its length, course, and site of insertion with CHD. Some variations which are clinically more important are the following: (i) low insertion of cystic duct, (ii) parallel course of cystic duct with CHD, (iii) anterior or posterior spiral course with medial insertion, (iv) absent or short cystic duct (length < 5 mm), (v) aberrant drainage of cystic duct to right hepatic or left hepatic duct, (vi) aberrant or accessory intrahepatic ducts draining into cystic duct, and (vii) double cystic duct [2–4].

Purpose of our study was to demonstrate the imaging features of various anatomical variants of cystic duct using magnetic resonance cholangiopancreatography (MRCP) and to document the prevalence of cystic duct variations in our population.

## 2. Materials and Method

This observational retrospective study was conducted after approval was obtained from the institutional research and ethics committee. All consecutive patients who underwent MRCP in our hospital for different indications over a period of one and half year, from July 2011 to Dec 2012, were included in our study. A total of 224 cases were evaluated among which cystic duct insertion was seen in 198 (88.4%) cases. Nine cases (4%) showed postcholecystectomy status and in 17 (7.6%) cases cystic duct insertion was not made out due to ductal pathology or overlapping of structures.

Imaging was performed in 1.5-Tesla MRI units (Achieva SE, Philips Healthcare) using a torso phased-array coil. Two MRCP sequences were used. The first was single-shot radial MRCP (TR/TE, 8000/800 ms; echo-train length, 256; flip angle, 90°; FOV, 300 mm^2^; section thickness, 40 mm; sections passing through the porta hepatis and rotating around a point anterior to the portal vein). The first coronal oblique image was through the tail of the pancreas, the second image was a straight coronal image, and subsequent sections were 15° apart. The second sequence was an MRCP high-resolution sensitivity encoding (SENSE) sequence (TR/TE, 1204/650; flip angle, 90°; FOV, 260 mm^2^; section thickness, 1 mm; interval, 0.8 mm; straight coronal sections). Maximum-intensity-projection sets of MRCP high-resolution SENSE sequence images were generated in the coronal plane.

### 2.1. Image Analysis

The MRCP images were assessed in PACS (Novarad). The length, course, and insertion of cystic duct were documented. When cystic duct joins the CHD at its upper third it was defined as high insertion and when it joins CHD at lower third it was defined as low insertion. Point of insertion was documented as lateral (to the right of CHD), anterior, posterior, and medial (to the left of CHD). Short cystic duct was defined as cystic duct length of less than 5 mm. Long parallel insertion was defined as parallel course of cystic duct with CHD for at least 2 cm.

*Statistical Methods*. Descriptive and inferential statistical analysis has been carried out in the present study. Results on continuous measurements are presented as mean ± SD (min–max) and results on categorical measurements are presented as number (%).

## 3. Results

Among 198 patients, 105 (53%) cases were male patients and 93 (47%) were female patients (mean age, 44 years; range, 12–78 years). The anatomical variations of cystic duct are summarized in Table 1.

**Figure 1:**
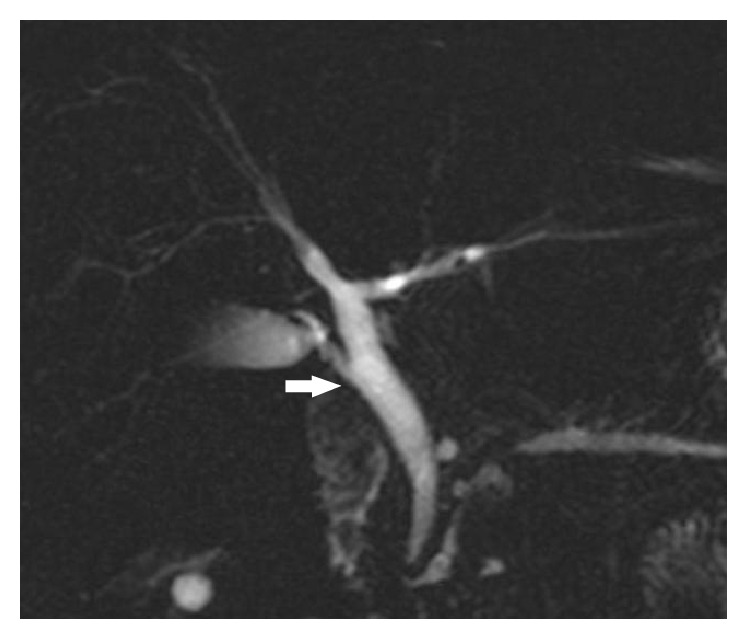
Coronal oblique 3D MR cholangiopancreatography shows normal insertion of cystic duct at middle 3rd of common hepatic duct from lateral aspect (arrow).

**Figure 2:**
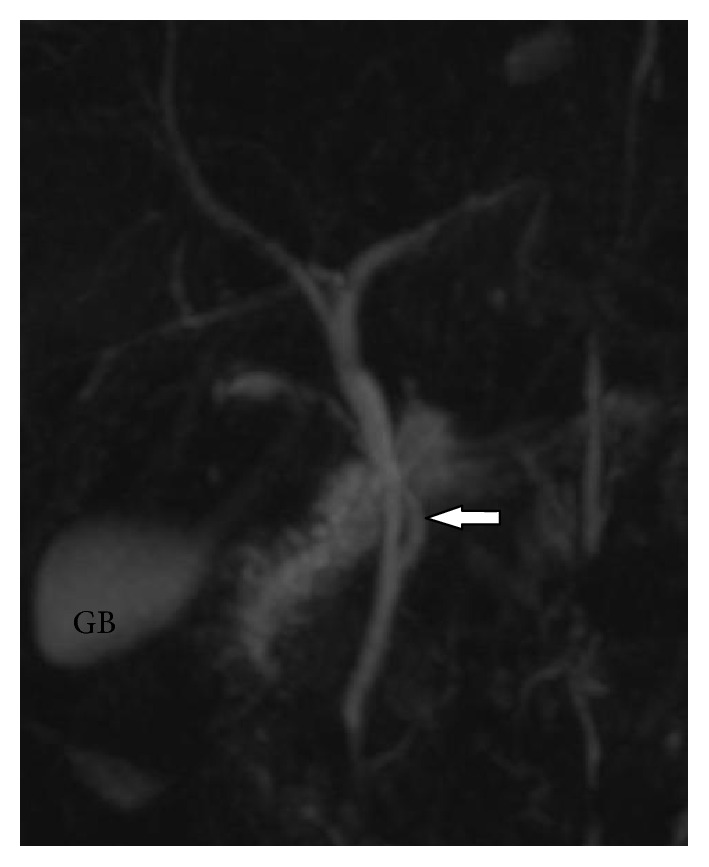
Coronal oblique 3D MR cholangiopancreatography shows spiral course of cystic duct (white arrow) with medial insertion with CHD. GB: gall bladder.

**Figure 3:**
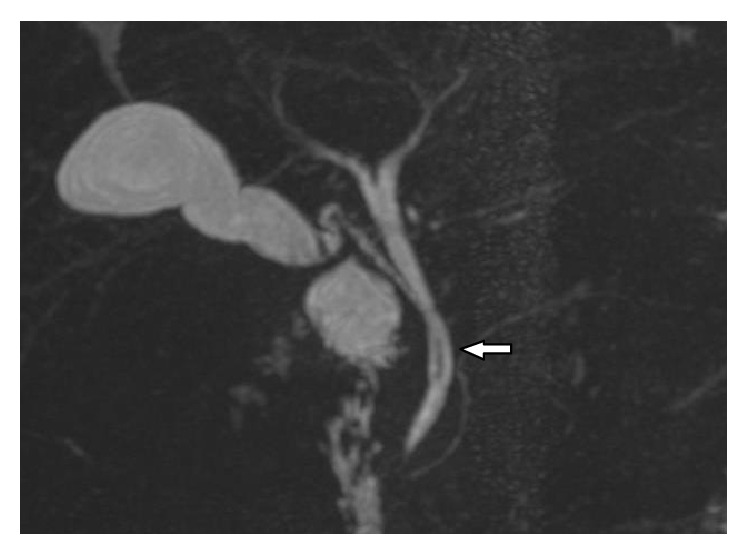
Coronal oblique 3D MR cholangiopancreatography shows low medial insertion of cystic duct where cystic duct (arrow) drains at lower 3rd of CHD from left side.

**Figure 4:**
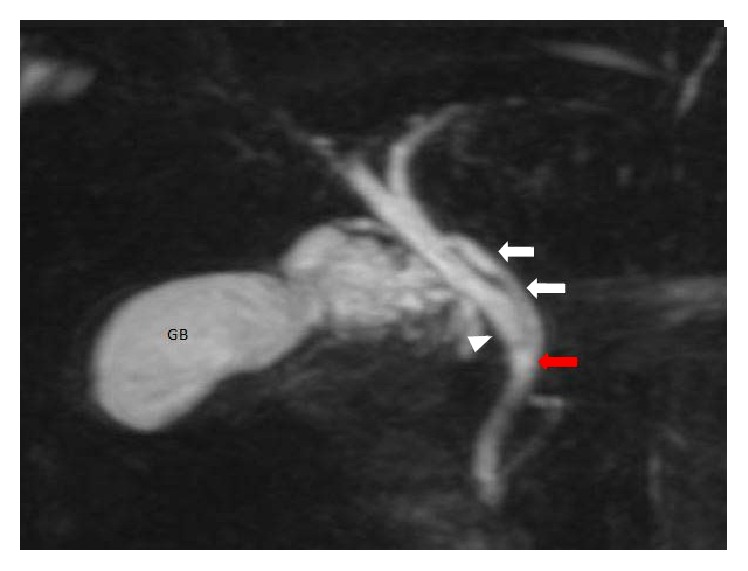
Coronal oblique 3D MR cholangiopancreatography shows parallel course of cystic duct (white arrow) and CHD (white arrowhead). Also note medial insertion of cystic duct (red arrow). GB: gall bladder.

**Figure 5:**
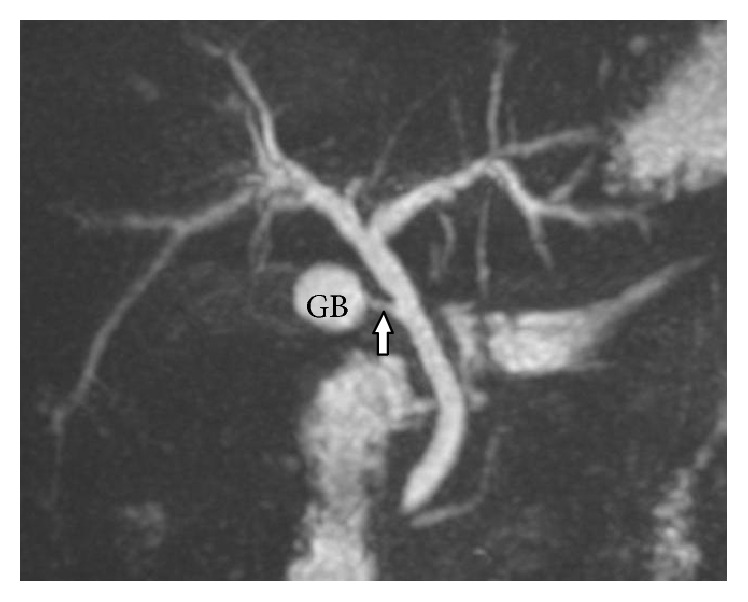
Coronal oblique 3D MR cholangiopancreatography shows high insertion of cystic duct at upper 3rd of CHD from lateral aspect.

**Figure 6:**
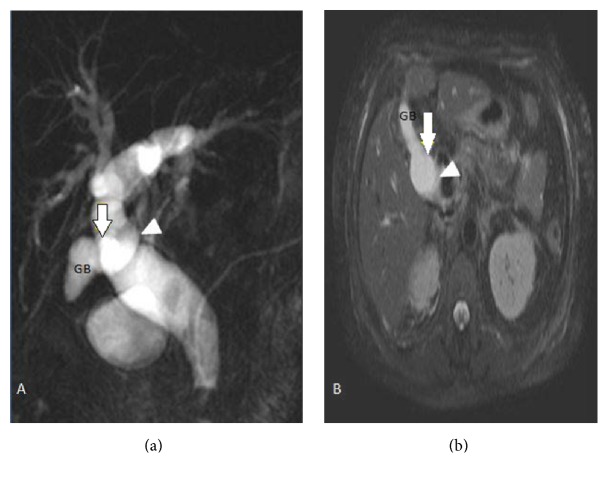
(a) Coronal oblique 3D MR cholangiopancreatography. (b) SSH SPAIR transverse image shows short cystic duct with anterior insertion (arrow) into the CHD (arrowhead). GB: gall bladder.

**Figure 7:**
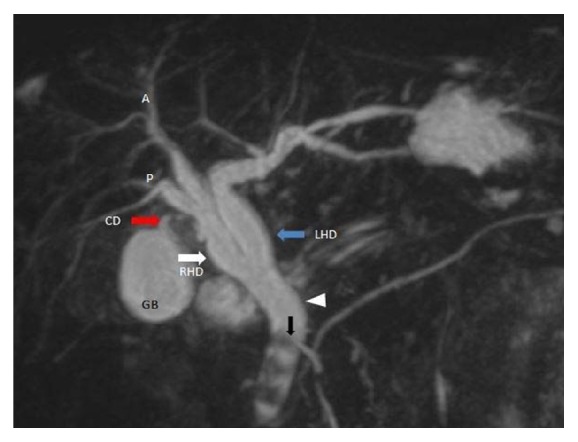
Coronal oblique 3D MR cholangiopancreatography shows aberrant insertion of cystic duct (red arrow) into the right hepatic duct (white arrow) and low union of right and left hepatic duct (blue arrow). Also note multiple calculi (black arrow) in common bile duct (white arrowhead). A: right anterior sectoral duct, P: right posterior sectoral duct, RHD: right hepatic duct, LHD: left hepatic duct, CD: cystic duct, and GB: gall bladder.

**Figure 8:**
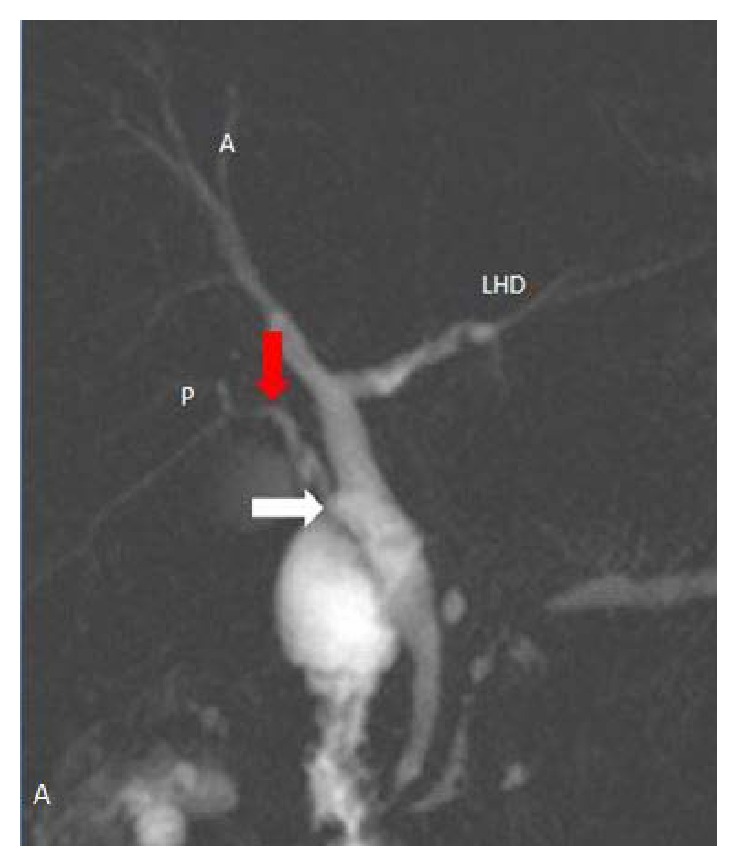
Coronal oblique 3D MR cholangiopancreatography shows aberrant union of right segmental bile duct (red arrow) into the cystic duct (white arrow). Cystic duct (white arrow) unites laterally to form CBD. LHD: left hepatic duct.

In 102 (51.5%) cases, normal lateral insertion of cystic duct at middle third of CHD was seen (Figure 1). Spiral course with medial insertion of cystic duct is seen in 32 (16.1%) cases (Figure 2). Low insertion of cystic duct was noted in 18 (9%) cases, in which 8 (4%) cases had low medial insertion (Figure 3). Parallel course of cystic duct was present in 15 (7.5%) cases (Figure 4). High insertion of cystic duct was noted in 11 (5.5%) cases (Figure 5). Short cystic duct was seen in 2 (1%) cases (Figure 6). In 2 of our cases, cystic duct was draining into the RHD (Figure 7). In these cases there was absence of CHD with low confluence of RHD and LHD. Aberrant right posterior sectoral bile duct draining into cystic duct is noted in 1 of our cases (Figure 8).

**Figure 9:**
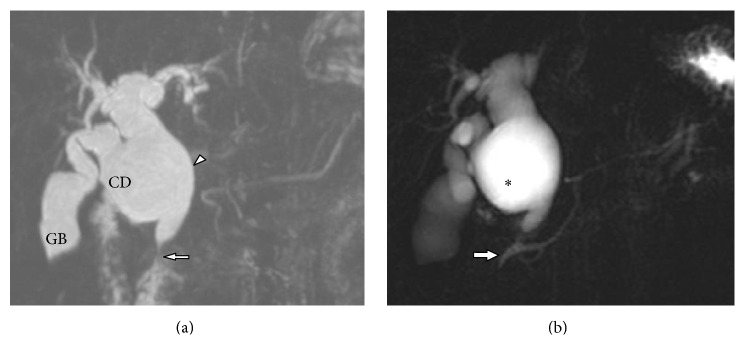
42-year-old female with choledochal cyst involving the cystic duct. (a) Coronal oblique 3D MR cholangiopancreatography shows marked fusiform dilatation of the extrahepatic bile duct (white arrowhead) with abrupt tapering at distal part (white arrow). The cystic duct is elongated and tortuous and showing cystic dilatation at its distal end with wide communication with the CHD. Also note that there was fusiform dilatation of left hepatic duct. (b) Coronal oblique 3D MR cholangiopancreatography shows abnormal union of pancreatobiliary duct with long common channel. *∗*: choledochal cyst. GB: gall bladder and CD: cystic duct.

Congenital cystic dilation of cystic duct was noted in one case with evidence of Todani type IV choledochal cyst (Figure 9). There was cystic dilatation of the CHD and proximal CBD with abrupt tapering at distal intrapancreatic part of CBD and mild focal dilatation of the right posterior segmental branch. The cystic duct is elongated and tortuous and showing cystic dilatation at its distal end. Wide communication is noted between cystic duct and CHD. There was anomalous pancreatobiliary duct union with long common channel.

## 4. Discussion

Bile duct injury is a serious complication during cholecystectomy, more commonly seen in laparoscopic cholecystectomy. One of the major causes of bile duct injury is failure to identify the ductal anatomy, particularly in the presence of anatomical variants. Complete transection of common bile duct occurs when CBD is mistaken for cystic duct and it is one of the dreaded complications of laparoscopic and open cholecystectomy [5]. Intraoperative cholangiography (IOC) is commonly performed during cholecystectomy to document the bile duct anatomy. In one series IOC was conclusive only in 57% of cases. Incomplete filling of bile duct and projection of cystic duct over CBD have resulted in false or inconclusive results [5].

MRCP is a noninvasive imaging modality which can optimally image the bile ducts and cystic duct. Studies have shown that preoperative MRCP provides important information regarding cystic duct anatomy and has a significant safeguarding effect on laparoscopic cholecystectomy [6–8]. Prior knowledge of the cystic duct anatomy and its variants helps in proper interpretation of disease process and avoids iatrogenic injuries. Preoperative documentation of bile duct anatomy may also help in medicolegal purposes [5].

Recently, percutaneous transcholecystic biliary interventions are being performed through the cystic duct. Prior knowledge of cystic duct anatomy and variations would definitely help in planning the procedure and avoiding complications [9].

Extreme variability is noted in the course of cystic duct and its junction with extrahepatic bile duct. Classical anatomy of cystic duct joining the CHD at its middle third from lateral aspect is seen in 58%–75% of cases [10]. We have seen this anatomy in 51.5% of our cases. The three most common and clinically significant variants are medial insertion of cystic duct, low insertion of cystic duct, and parallel course of cystic duct.

16% of our cases revealed medial insertion with posterior or anterior spiral course. Medial insertion of cystic duct was reported in 10–18% of cases in previous studies [11–13]. This variant is important during surgery. Dissection of the medial cystic duct up to its end is considered dangerous and it is advisable to leave a long remnant of cystic duct [14].

Low insertion of cystic duct (LICD) was reported in 8 to 11% of cases in previous studies [11, 15, 16]. 9% of our cases showed LICD, among which 4% had low medial insertion. Low insertion of cystic duct was associated with high rate of CBD stone formation and higher recurrence of CBD stones [16, 17]. Failure to identify a low insertion of cystic duct may result in technical difficulties during ERCP procedures and may lead to complication [18].

A long parallel CHD and cystic duct were reported in 1.2–25% of the population, where these ducts are surrounded by a common fibrous sheath and show parallel course for at least 2 cm [12, 14]. This variation was noted in 7.5% of our cases. If this variant is not recognized, the extrahepatic bile duct can be mistaken as the cystic duct and can result in inadvertent section or ligation of the extrahepatic bile duct and lead to postoperative complication. If the long parallel cystic duct is ligated or transected too close to the CHD, the CHD can undergo strictures or narrowing at this site. In patient with long parallel cystic duct and cases with medial insertion, usually long cystic duct is left after cholecystectomy. This is more frequently associated with inflammatory changes and calculus disease leading to postcholecystectomy syndrome [1].

The presence of short or absent cystic duct is a rare but important variant and increases the chance of biliary injury, especially during laparoscopic cholecystectomy [19]. Short cystic duct was reported in 1.3%–2.6% of cases in previous studies [10, 11, 20]. This anomaly was noted in two of our cases (1%). During surgery when surgeons try to visualize the cystic duct by giving traction on gall bladder, presence of short cystic may result in tenting of the CHD or CBD and inadvertent clamping of these ducts [20].

Aberrant drainage of cystic duct into right hepatic duct is rare and reported in 0.3%–0.4% of patients [21]. We have also seen 2 cases in which cystic ducts were draining into the RHD and one case of aberrant intrahepatic duct draining into cystic duct. It is crucial to diagnose the high union of the cystic duct into the CHD, aberrant cystic duct drainage into the right hepatic duct, and aberrant union of intrahepatic bile ducts to the cystic duct as these variants can be misdiagnosed during surgery, leading to inadvertent transaction and ligation.

We have not seen any case of double cystic duct in our study. Cystic duct duplication in the presence of single gall bladder is a very rare anomaly and is associated with higher risk of complication during laparoscopic cholecystectomy. This anomaly can be confused with accessory intrahepatic duct draining into the CHD. Preoperative or intraoperative cholangiogram is very crucial in proper identification of these variations and avoiding complications [3, 22].

Choledochal cyst is congenital dilatation of intrahepatic and extrahepatic bile ducts and classified by Todani et al. into five types [23]. Choledochal cyst involving the cystic duct was not described in this classification. However, several case reports and case series have reported isolated cystic malformation of cystic duct and cystic dilatation of cystic duct associated with other types of choledochal cysts [24–26].

We have also seen one case of type IV choledochal cyst associated with fusiform dilatation of cystic duct. Awareness of this type of malformation of the cystic duct would help in correct preoperative diagnosis and appropriate treatment strategy. MRCP is the preferred imaging modality for diagnosis of this condition which clearly delineates the anatomy and relationship of entire biliary tract. It can simultaneously delineate the abnormal union of pancreatobiliary duct which is reported in 33–90% of cases [27].

The limitation of our study is that we could not compare our results with ERCP or intraoperative cholangiography. We could not evaluate cystic duct in all patients due to adjacent ductal pathology or overlapping of structures.

## 5. Conclusion

Cystic duct variations are not uncommon and it is important to recognize the anatomical variations. MRCP is an excellent imaging modality for demonstration of cystic duct anatomy and its variations which not only helps in proper interpretation of the disease process but also provides a road map before any percutaneous, endoscopic, and surgical interventions.

## Figures and Tables

**Table 1:** Distribution of different anatomical variations of cystic duct.

Type of cystic duct variations	Frequency *n* = 198	%
(1) Spiral course with medial insertion	32	16.1
(2) Low insertion	18	9
(3) Low medial insertion	8	4
(4) High insertion	11	5.5
(5) Anterior insertion	4	2
(6) Posterior insertion	40	20.2
(7) Parallel course of cystic duct	15	7.5
(8) Short cystic duct	2	1
(9) Cystic duct draining to right hepatic duct	1	0.5
(10) Right posterior sectoral hepatic duct draining to cystic duct	1	0.5

## References

[1] Turner M. A., Fulcher A. S. (2001). The cystic duct: normal anatomy and disease processes. *RadioGraphics*.

[2] Wu Y.-H., Liu Z.-S., Mrikhi R. (2008). Anatomical variations of the cystic duct: two case reports. *World Journal of Gastroenterology*.

[3] Fujikawa T., Takeda H., Matsusue S., Nakamura Y., Nishimura S. (1998). Anomalous duplicated cystic duct as a surgical hazard: report of a case. *Surgery Today*.

[4] Hashimoto M., Hashimoto M., Ishikawa T., Iizuka T., Matsuda M., Watanabe G. (2002). Right hepatic duct emptying into the cystic duct: report of a case. *Surgical Endoscopy*.

[5] Buddingh K. T., Morks A. N., Ten Cate Hoedemaker H. O. (2012). Documenting correct assessment of biliary anatomy during laparoscopic cholecystectomy. *Surgical Endoscopy*.

[6] Zhang C., Yin M., Liu Q. (2015). The guidance impact of preoperative magnetic resonance cholangiopancreatography on laparoscopic cholecystectomy. *Journal of Laparoendoscopic & Advanced Surgical Techniques Part A*.

[7] Itatani R., Namimoto T., Kajihara H. (2013). Preoperative evaluation of the cystic duct for laparoscopic cholecystectomy: comparison of navigator-gated prospective acquisition correction- and conventional respiratory-triggered techniques at free-breathing 3D MR cholangiopancreatography. *European Radiology*.

[8] Ausch C., Hochwarter G., Taher M. (2005). Improving the safety of laparoscopic cholecystectomy: the routine use of preoperative magnetic resonance cholangiography. *Surgical Endoscopy and Other Interventional Techniques*.

[9] Hatzidakis A., Venetucci P., Krokidis M., Iaccarino V. (2014). Percutaneous biliary interventions through the gallbladder and the cystic duct: what radiologists need to know. *Clinical Radiology*.

[10] Talpur K. A. H., Laghari A. A., Yousfani S. A., Malik A. M., Memon A. I., Khan S. A. (2010). Anatomical variations and congenital anomalies of extra hepatic biliary system encountered during laparoscopic cholecystectomy. *Journal of the Pakistan Medical Association*.

[11] Önder H., Özdemir M. S., Tekbaş G., Ekici F., Gümüş H., Bilici A. (2013). 3-T MRI of the biliary tract variations. *Surgical and Radiologic Anatomy*.

[12] Shaw M. J., Dorsher P. J., Vennes J. A. (1993). Cystic duct anatomy: an endoscopic perspective. *American Journal of Gastroenterology*.

[13] Mortelé K. J., Ros P. R. (2001). Anatomicvariants of the biliary tree: MR cholangiographic findings and clinical applications. *American Journal of Roentgenology*.

[14] Mortelé K. J., Rocha T. C., Streeter J. L., Taylor A. J. (2006). Multimodality imaging of pancreatic and biliary congenital anomalies. *Radiographics*.

[15] Taourel P., Bret P. M., Reinhold C., Barkun A. N., Atri M. (1996). Anatomic variants of the biliary tree: diagnosis with MR cholangiopancreatography. *Radiology*.

[16] Tsitouridis I., Lazaraki G., Papastergiou C., Pagalos E., Germanidis G. (2007). Low conjunction of the cystic duct with the common bile duct: does it correlate with the formation of common bile duct stones?. *Surgical Endoscopy and Other Interventional Techniques*.

[17] Kao J.-T., Kuo C.-M., Chiu Y.-C., Changchien C.-S., Kuo C.-H. (2011). Congenital anomaly of low insertion of cystic duct: endoscopic retrograde cholangiopancreatography findings and clinical significance. *Journal of Clinical Gastroenterology*.

[18] George R. A., Debnath J., Singh K., Satija L., Bhargava S., Vaidya A. (2009). Low insertion of a cystic duct into the common bile duct as a cause for a malpositioned biliary stent: demonstration with multidetector computed tomography. *Singapore Medical Journal*.

[19] Selvaggi F., Cappello G., Astolfi A. (2010). Endoscopic therapy for type B surgical biliary injury in a patient with short cystic duct. *Il Giornale di Chirurgia*.

[20] Awazli L. G. (2013). Anatomical variations of extrahepatic biliary system. *Iraqi Journal of Medical Science*.

[21] Carbajo M. A., Martín del Omo J. C., Blanco J. I. (1999). Congenital malformations of the gallbladder and cystic duct diagnosed by laparoscopy: high surgical risk. *Journal of the Society of Laparoendoscopic Surgeons*.

[22] Tsutsumi S., Hosouchi Y., Shimura T. (2000). Double cystic duct detected by endoscopic retrograde cholangiopancreatography and confirmed by intraoperative cholangiography in laparoscopic cholecystectomy: a case report. *Hepato-Gastroenterology*.

[23] Todani T., Watanabe Y., Narusue M., Tabuchi K., Okajima K. (1977). Congenital bile duct cysts: classification, operative procedures, and review of thirty-seven cases including cancer arising from choledochal cyst. *The American Journal of Surgery*.

[24] Yoon J.-H. (2011). Magnetic resonance cholangiopancreatography diagnosis of choledochal cyst involving the cystic duct: report of three cases. *British Journal of Radiology*.

[25] Maheshwari P. (2012). Cystic malformation of cystic duct: 10 cases and review of literature. *World Journal of Radiology*.

[26] Sethi S., Upreti L., Verma A. K., Puri S. K. (2015). Choledochal cyst of the cystic duct: report of imaging findings in three cases and review of literature. *Indian Journal of Radiology and Imaging*.

[27] Lee H. K., Park S. J., Yi B. H., Lee A. L., Moon J. H., Chang Y. W. (2009). Imaging features of adult choledochal cysts: a pictorial review. *Korean Journal of Radiology*.

